# Imaging of cardiovascular risk in patients with Turner's syndrome

**DOI:** 10.1016/j.crad.2015.03.009

**Published:** 2015-08

**Authors:** A. Marin, J.R. Weir-McCall, D.J. Webb, E.J.R. van Beek, S. Mirsadraee

**Affiliations:** a Clinical Research Imaging Centre, Queen's Medical Research Institute, University of Edinburgh, Edinburgh EH16 4TJ, UK; b Division of Cardiovascular and Diabetes Medicine, Ninewells Hospital & Medical School, Dundee DD1 9SY, UK; c Queen's Medical Research Institute, University of Edinburgh/BHF Centre for Cardiovascular Science, Edinburgh EH16 4TJ, UK

## Abstract

Turner's syndrome is a disorder defined by an absent or structurally abnormal second X chromosome and affects around 1 in 2000 newborn females. The standardised mortality ratio in Turner's syndrome is around three-times higher than in the general female population, mainly as a result of cardiovascular disorders. Most striking is the early age at which Turner's syndrome patients develop the life-threatening complications of cardiovascular disorders compared to the general population. The cardiovascular risk stratification in Turner's syndrome is challenging and imaging is not systematically used. The aim of this article is to review cardiovascular risks in this group of patients and discuss a systematic imaging approach for early identification of cardiovascular disorders in these patients.

## Introduction

Turner's syndrome (TS) or Ullrich–Turner's syndrome is a disorder defined by an absent or structurally abnormal second X chromosome and affects around 1 in 2000 newborn females.^1^ The variable phenotypes can be split into three main categories: monosomy X karyotype (45,X) (in 36–45%); mosaic karyotype (44–54%); and an isochromosome Xq, (5–11%).^2,3^ Short stature, gonadal dysgenesis, and congenital cardiovascular defects are common features of TS.^4^ Congenital heart defects, such as hypoplastic left heart syndrome and/or hypoplastic aortas, are the major causes of prenatal mortality.^5–7^ The foetuses with less severe cardiovascular defects survive the first trimester and can be recognised by *in-utero* ultrasound by hydrops of the trunk and limbs, large and loculated cystic hygromas of the posterolateral neck, pleural effusions, and ascites.^6^ When these resolve they leave the postnatal webbing of the neck (pterygium colli), puffy hands and feet, or redundant nuchal skin, making the diagnosis possible in 20–30% of newborn girls with TS.^4^ Around one-third are diagnosed in mid-childhood on the investigation of short stature and broad chest. In most other patients, who have milder signs and symptoms of TS, the condition is diagnosed due to delayed or absent pubertal development secondary to gonadal dysgenesis either in adolescence or in adulthood.^4^ The most common cardiovascular defects in surviving TS patients are bicuspid aortic valve (BAV) and aortic coarctation. These patients are also at increased risk for hyperlipidaemia, hypertension, and atherosclerosis.^8^

**Figure 1:**
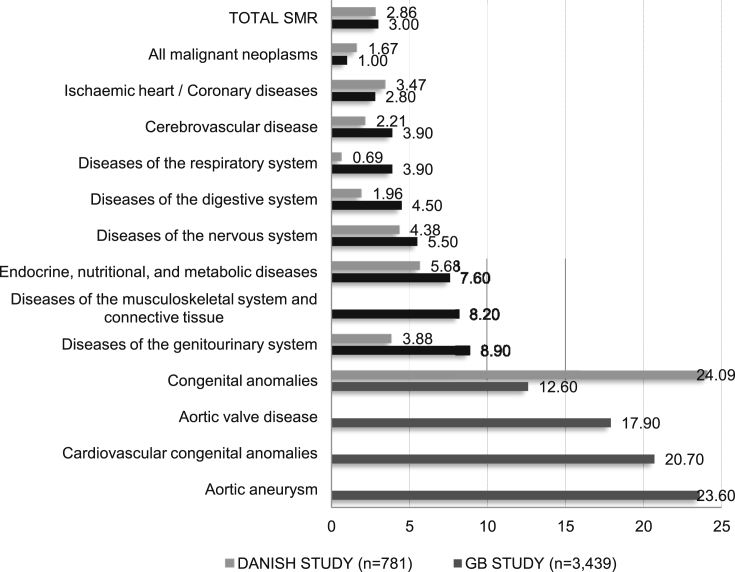
Total and cause-specific SMR in Turner's syndrome by main diagnostic groups, from the Denmark^2^ and Great Britain^3^ cohort studies data. SMR is the ratio of deaths in the study population compared with the number expected from rates in the general population. The Danish study used the ICD-10, while the study from Great Britain used ICD-9 for the classification of diagnostic groups. Some cause-specific diagnostic subcategories (e.g., diseases of musculoskeletal system, aortic valve disease, cardiovascular congenital anomalies and aortic aneurysm) were not separately included in the Danish cohort study data. The high congenital anomalies SMR in Danish cohort is likely attributable to malformations of the heart and great arterial vessels.

The age-specific death rate in TS is around three-times higher than in the general female population with Fig 1 tabulating the relative standardised mortality ratios (SMRs).^2,3^ Cardiovascular disease accounts for 41% of excess deaths and the relative risk is most markedly elevated with cardiovascular congenital anomalies; in particular BAV and aortic aneurysm.^3^ The risk of acquired aortic dissection is increased by up to 100-fold and can occur in TS patients as young as 16–18 years.^9,10^

The above highlights the need for appropriate cardiovascular risk stratification in TS patients. In the UK and some other countries, dedicated TS clinics have been established where teams of paediatric endocrinologists, gynaecologists, cardiologists, radiologists, and hypertension specialists are implementing appropriate screening and management strategies.^8,11^ The aim of this paper is to review the role of clinical imaging in TS cardiovascular risk stratification.

## Methodology

### Literature search

Publications were identified by a systematic literature search using PubMed to identify studies evaluating medical issues in TS published between January 1990 and August 2014. The search terms used in the Medical Subject Headings (MeSH) Database were "Turner Syndrome/complications"[Mesh] OR "Turner Syndrome/etiology"[Mesh] OR "Turner Syndrome/mortality"[Mesh] OR "Turner Syndrome/radiography"[Mesh] OR "Turner Syndrome/ultrasonography"[Mesh] OR ″Turner Syndrome/imaging"[Mesh] OR "Turner Syndrome/cardiovascular"[Mesh] OR "Turner Syndrome/congenital"[Mesh]. Out of the 1242 papers found, 82 met the selection criteria. Only full-length original articles were included. Non-English texts, experimental studies, and case series with fewer than five patients were excluded. In addition, the references were revised and eligible articles that were not captured by the search strategy were identified.

## Congenital cardiovascular disorders and complications

Cardiovascular anomalies (Table 1) are present in up to 50% of the TS population and are the major cause of premature mortality.^3,9,10,12–15^ The most commonly occurring cardiovascular anomalies are a BAV, aortic dilatation, elongation of the thoracic arch, aortic coarctation, and partial anomalous pulmonary venous return.

### Pathogenesis

The pathogenesis of these cardiovascular defects is still unclear. Previously, it was thought that the left heart outflow tract defects were caused by increased fetal lymphatic pressure and jugular lymphatic sac obstruction leading to obstruction to or reduction of the blood flow within the developing heart, resulting in the observed left heart defects. This hypothesis also encompassed the development of anomalous pulmonary venous drainage due to the hold-up of blood flow within the pulmonary bed secondary to the left heart defects.^16^ However, more recently published papers have demonstrated the presence of aortic coarctation and BAVs in TS without evidence of fetal lymphoedema, and that the presence of partial anomalous pulmonary venous return is not associated with either aortic coarctation or BAV, both observations in contradiction to the original hypothesis.^17,18^ Miyabara^19^ and colleagues suggest an alternative idea, that a primary neural crest defect in the region responsible for the formation of the 4^th^ pharyngeal pouch and 4^th^ branchial branch was responsible for both the congenital heart and lymphatic anomalies. Regardless of the exact embryological trigger for the developmental anomalies in TS, presuming a single trigger can account for all of the anomalies, great leaps have been made in the understanding of the genetic determinants of congenital heart defects. Cardiovascular defects, aortic aneurysm, and dissection are most frequently observed among TS patients with 45,X karyotype, with a high prevalence of congenital heart defects also present in TS females missing only the X chromosome short arm, meaning that haplo-insufficiency for Xp genes contributes to the abnormal aortic valve and aortic arch development in TS.^3,18,20^ In the future, this knowledge may allow a more targeted identification of those most likely to benefit from a more detailed or intensive investigation programme.

### Clinical presentations

**Figure 2:**
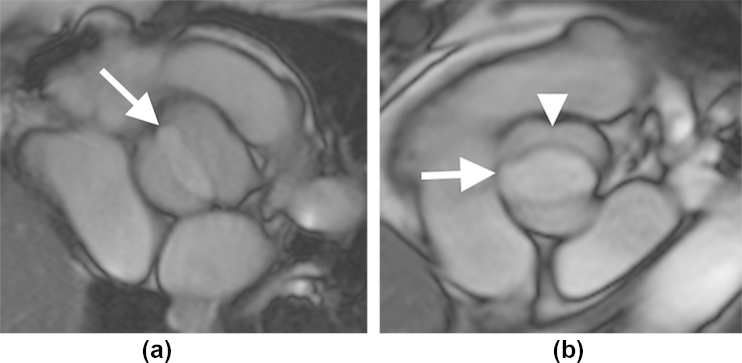
Systolic cine MRI of the BAV in two patients with TS. (a) Demonstrates a R-L BAV (arrow) and (b) demonstrates a R-L BAV (arrow) with raphe (arrowhead).

**Figure 3:**
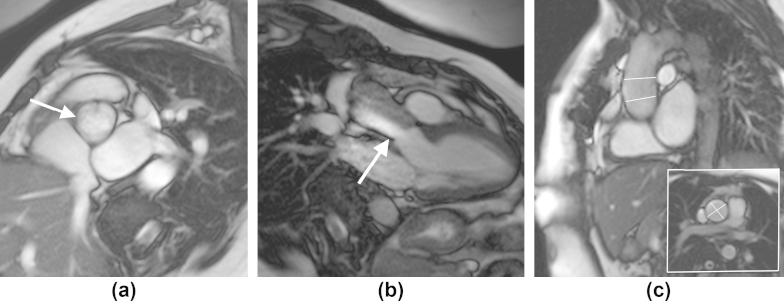
Systolic bSSFP cine MRI of the aortic valve (a), the aortic root (b) and ascending aorta (c,d) in a 45-year-old TS patient with mild aortic stenosis and aortic regurgitation on transthoracic echocardiography, which was unable to assess the aortic dimensions. (a) Atrioventricular view demonstrates a L-R BAV (arrow), and (b) the three-chamber view shows an asymmetric jet flow through the BAV (arrow). (c) Shows the aortic root and ascending aorta in sagittal oblique (diameter measurements white lines) and left bottom figure (white frame) in axial views at the level of the main pulmonary artery.

BAV is the most common congenital cardiovascular malformation occurring in up to 30% of TS patients compared with just 1–2% in the general population (Fig 2).^21,22^ Individuals with BAV are at increased risk of aortic coarctation and/or aortic dilatation.^22^ BAV at a young age is usually clinically silent, but with age BAV tends to more rapidly degenerate and calcify, resulting in progressive stenosis and/or regurgitation (Fig 3).^23,24^ In 95% of adult TS females, BAV is a consequence of fusion of the right and left coronary leaflets (R-L BAV), while the fused right coronary and non-coronary leaflets (R–NC BAV) variant is much less common.^22^ However, a single study of post-mortem heart specimens from 36 TS fetuses and one TS newborn reported a larger proportion of R-NC BAV type (31%).^25^ The pattern of aortic valve leaflet fusion in patients with BAV may be important, because general population studies have demonstrated that the R-NC BAV is associated with a higher prevalence of significant aortic valve stenosis and regurgitation, whereas the R-L BAV is associated with aortic coarctation, dilatation, and less frequently with aortic valve pathology.^26,27^ Both a genetic and flow-mediated hypothesis have been proposed for the association between BAV and aortopathy.^28^ The genetic model proposes that a disorder in one of the genes responsible for vascular connective tissue development is responsible for both BAV formation and the subsequent propensity for aortic dilatation. However, a growing body of evidence demonstrates an abnormal eccentric flow pattern, which is directed at the lateral ascending aortic wall in those with R-L fusion, resulting in wall remodelling secondary to elevated regional wall shear stress even where there is no significant transvalvular gradient or regurgitation.^29–31^

The role of imaging in BAV is for the detection of fused leaflets, the characterisation of the location of the fusion and the quantification of valvular function. Transthoracic two-dimensional and colour Doppler echocardiography enables a reliable non-irradiating assessment of the cardiovascular anatomy including the aortic valve and root and the ascending part of the thoracic aorta, as well as providing accurate assessment of valvular function.^32^ However, transthoracic echocardiography can be inadequate in visualising the aortic valve in TS in around 6%, with BAV itself as a risk factor for non-visualisation of the aortic valve.^33^ Other factors for suboptimal visualisation of the aortic valve with transthoracic echocardiography are calcification of the valve and a poor acoustic window (associated with obesity and short stature, both common occurrences in women with TS).^34,35^ In these instances, cardiac MRI is useful for further evaluation of the anatomy of the valve using balanced steady-state free precession (bSSFP) cine sequence to visualise the valve, with phase-contrast MRI for functional assessment. MRI typically underestimates the severity of aortic stenosis compared with echocardiography due to flow vorticity causing signal loss. However, MRI is associated with less inter-scan and interobserver variability.^36–38^

**Figure 4:**
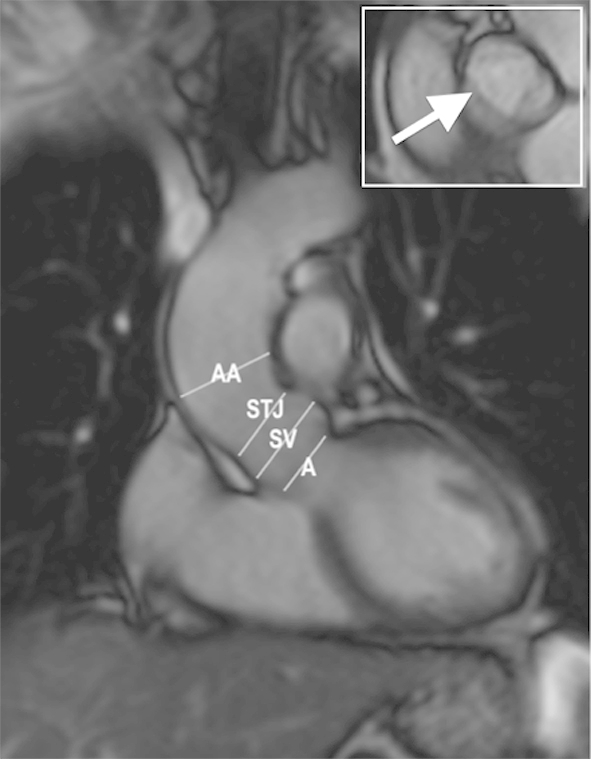
An oblique coronal left ventricular outflow tract cine image (bSSFP) end diastolic frame of a 50-year-old patient with TS. Right top figure (white frame) shows a normal tricuspid aortic valve (white arrow) in this patient. Measurements of the annulus (A), sinus of Valsalva (SV), sinotubular junction (STJ), and ascending aorta (AA) were within the normal limits for an adult patient: 20, 27, 22, and 32mm, respectively. However, the calculated ASI was 2.28 cm/m^2^ (body surface area = 1.4m^2^) indicating a higher risk of future growth and/or dissection.

Aortic dilatation is reported in 32–42% of women with TS.^15,39,40^ Table 2 summarises the studies on aortic dilatation in Turner's syndrome. Aortic dilatation may occur in isolation. However, it is most commonly found in association with BAV and/or aortic coarctation, with one study showing BAV to be present in 85% of those with aortic dilatation.^13,41^ Given that age and body size are strongly predictive of aortic diameter, use of standard absolute values for the evaluation and diagnosis of aortic dilatation in TS, with its abnormal body posture, is grossly inaccurate.^42^ Comparison with normograms derived from age-matched women is also flawed for the same reason.^43^ The use of an ascending/descending aortic diameter (AD/DD) ratio circumvents some of these issues as it provides an individualised normative value against which to compare the ascending aortic diameter. Using this measure, a ratio >1.5 is indicative of ascending aorta dilatation.^39^ However, this can be true only if the descending aorta diameter is normal. Another alternative, which correlates more closely with aortic diameter, is the use of the body surface area (BSA), and calculation of the ascending aortic size index (ASI) using the calculation: aortic diameter/BSA. The latter is more accurate in the assessment of aortic dilatation and the prediction of aortic dissection, with an ASI ≥2 cm/m^2^ considered as aneurysmal, with a higher risk of future growth and/or dissection requiring close surveillance (an example is the patient shown in Fig 4), while an ASI ≥2.5 cm/m^2^ poses an extremely high risk and a need for prompt surgical intervention.^5,40^ Recently, Mortensen et al.^44^ have derived a mathematical model that uses current aortic dimensions, presence of BAV, aortic coarctation, diastolic blood pressure, and BSA to predict those with a high risk of rapid progression of aortic dilatation.^44^ However, this has yet to be externally validated in a prospective study.

MRI is inarguably the optimal method for assessment of the thoracic aorta. However, the precise choice of technique varies from centre to centre. A 2007 guideline on the management of TS suggested that the aorta should be measured in end-systole. This is readily achievable using a black blood turbo spin-echo (TSE) sequence, although this suffers from only measuring a single point of the aorta, and changes in slice angulation can lead to overestimation of diameter. Alternatively, a respiratory navigated, electrocardiogram (ECG)-gated three-dimensional (3D) steady-state free-procession (bSSFP) technique will provide images of the entire thoracic aorta in diastole, allowing accurate measurement of the aortic root as well as the ascending and descending aorta, although this comes at a cost of a significantly prolonged acquisition time.^45^

The data underpinning our understanding of aortic dissection in TS is extremely limited (Table 2). What is well established is that TS is associated with an elevated risk of developing aortic dissection and that dissections occur at a much earlier age. The incidence of aortic dissection is estimated to be as much as 100-fold higher in TS compared to the general population, although this value was based on only three dissections in a population of 166 females with TS.^40^ A more conservative estimate is a six-times increase in relative risk in a study by Gravholt et al.,^9^ although this compared the TS cohort with an unmatched population rate and is, therefore, likely extremely conservative. Most striking is the early age at which TS patients develop dissection, with a mean age of 30.5 years (interquartile range [IQR] 23.5–38.5), compared with a mean age in the general female population of 77 years.^46^ It is estimated that at least 1.4% of females with TS will suffer an aortic dissection.^9^

One or more predisposing cardiovascular risk factors (BAV, aortic dilatation, and aortic coarctation) can be identified with cardiovascular imaging in up to 90% of aortic dissections in TS.^9,13,15^ Hypertension is also strongly associated with aortic dilatation and dissection, while upper extremity hypertension is a hallmark of significant aortic coarctation. However, aortic dilatation occurs at an early age in BAV and cannot thus be attributed solely to hypertension.^47^ Finally, pregnancy in TS appears to be associated with a high risk of dissection.^48^ In those who do dissect during pregnancy, prognosis is extremely poor with an 86% mortality in one series.^49^ Thus, careful cardiac and thoracic screening is essential in all those considering assisted contraception or pregnancy, with some experts advising an ASI >2 cm/m^2^ to be a contraindication to pregnancy.^50^

Identification and intervention in high-risk individuals is challenged by the difficulty in defining a dilated aorta as mentioned before. In a study by Matura et al.,^40^ all patients with dissection had an ASI >2.5, while two-thirds had AD:DD >1.5. In a study by Carlson et al.^46^ the mean ASI was 2.7 ± 0.6 cm/m^2^ in those with dissection, with two out of the 10 dissections occurring in patients with ASI <2.5 cm/m^2^, although the measurements were with echocardiography and taken up to 6 years prior to the dissection. In a study cohort of 166 TS patients, it was shown that dissection occurred in 25% with AD >3.5 cm, 33% of those with ASI >2.5, and only 3% with AD:DD >1.5, suggesting ASI as the most useful indicator of a dilated aorta with requirement for urgent intervention when a threshold of 2.5 cm/m^2^ is reached. Although MRI is the technique of choice in the screening and follow-up of those at risk for dissection, computed tomography (CT) angiography is typically the technique of choice in the acute assessment of those presenting with symptoms of dissection due to its wide availability and speed. Ideally, CT aortography should be performed with ECG gating due to improved visualisation of the proximal ascending aorta.^51^

**Figure 5:**
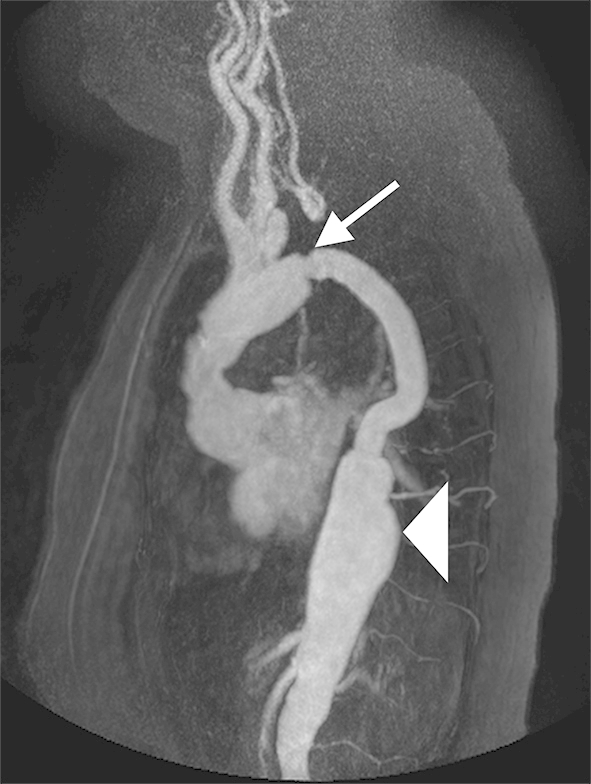
Volume-rendered MR aortography in a TS patient with previous aortic dissection and the residual coarctation (white arrow) and aortic aneurysm of descending thoracic aorta (white arrowhead) following surgical repair.

**Figure 6:**
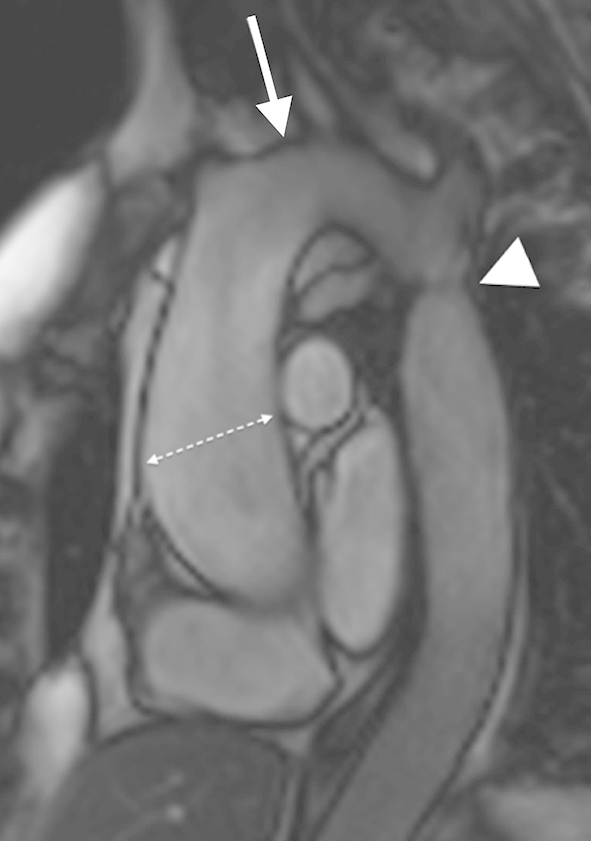
Sagittal oblique cine SSFP image of a 55-year-old TS patient showing a high-riding aorta (white arrow), residual coarctation after surgical repair (white arrowhead), and dilatation of ascending aorta (white dashed arrow), with the ASI >2.5.

Aortic coarctation affects 12% of women with TS.^12^ Additionally, elongated transverse arch and kinking of the isthmic portion of aorta can be seen in around half of all TS cases (Figs 5 and 6).^12,52^ This is likely part of a spectrum of abnormalities affecting the thoracic aorta, with elongated transverse arch as the mildest expression, and aortic arch hypoplasia/aplasia representing the most severe end of the spectrum, observed in around 2% of TS females.^52^ Aortic coarctation has been shown to be associated with dissection, and it has been proposed that elongated transverse arch may similarly predispose to dissection due to abnormal flow velocities and shear stress within the arch, although this has not been properly evaluated.^12^ Aortic coarctation is well evaluated with MRI, using either MR angiography (MRA) or a 3D bSSFP sequence, with both techniques showing a tight stenosis at the aortic isthmus with or without collateral formation. Current guidelines of European Society of Cardiology suggest intervention when there is >50% stenosis at the coarctation compared to the aortic diameter at the diaphragm with hypertension (Class IIa recommendation) with consideration to intervene even in the absence of hypertension (Class IIb recommendation).^53^ Additional functional assessment can be performed with velocity-encoded cine-MRI. Indicators of significant flow impairment include: greater flow through the distal thoracic aorta than through the thoracic aorta immediately distal to the coarctation; retrograde intercostal artery flow; and a diastolic tail with loss of the normal systolic–diastolic variation in flow. A pressure gradient across the stenosis can be calculated using the modified Bernoulli equation (Δ*P* = 4*v*^2^) where *v* is the maximum velocity through the coarctation. A gradient above 15 mmHg has been suggested as being significant on MRI, whereas echocardiography uses a threshold of 20 mmHg, which is similar to that used in invasive catheter measurements, although MRI often underestimates the peak velocity, hence the discrepancy.^53,54^ However, significant collateral flow can result in the pressure gradient being artificially low, and thus, quantification of collateral flow is considered a more accurate assessment of the haemodynamic significance of the stenosis.^55^

Partial anomalous pulmonary venous return is the most common venous anomaly and is found in 13% of TS cases, compared with ∼1% of the general population. This can present with right heart failure or pulmonary hypertension due to left to right shunting causing right ventricular and pulmonary volume overload.^56^ Careful scrutiny is important in cross-sectional imaging as visualisation of the pulmonary veins can be particularly challenging using transthoracic echocardiography.^57,58^ Persistent left-sided superior vena cava is also commonly found in TS (13%, compared to 1% in the general population). This is usually asymptomatic, but is important to report because it has the potential to cause confusion during central venous or right heart catheterisation, it may act as a route for paradoxical emboli when it inserts into the left atrium, and can cause complications in cardiothoracic surgery.^59^ A simple half-Fourier acquisition single-shot turbo spin-echo (HASTE) stack is often sufficient to visualise these anomalies. However, MRA or 3D bSSFP can also be useful to provide further visualisation of the vessels in case of uncertainty.

Other congenital cardiovascular disorders that can be observed in TS are ventricular septal defects, hypoplastic left heart syndrome, single ventricle, mitral valve abnormalities, atrial septal defects, coronary artery abnormalities, and aberrant right subclavian artery.^14^ In the retrospective review by Cramer et al.,^14^ two TS patients out of 173 had coronary artery abnormalities: one patient had an anomalous left coronary artery from the pulmonary artery, and the other patient had a small coronary artery fistula from the left coronary artery to the pulmonary artery. The risk for coronary artery disease is higher in TS women when compared to the general population.^60^ Women with TS more often have higher values of body mass index and waist:hip ratio, higher values of diastolic blood pressure, and higher levels of total cholesterol and low-density lipoprotein fraction, whereas levels of the high-density lipoprotein fraction are lower. Cardiac imaging in women with TS also reveals an increased left ventricular mass in association with aortic valve disease, age, hypertension, physical stature, and metabolic status.^61,62^

## The imaging techniques

**Figure 7:**
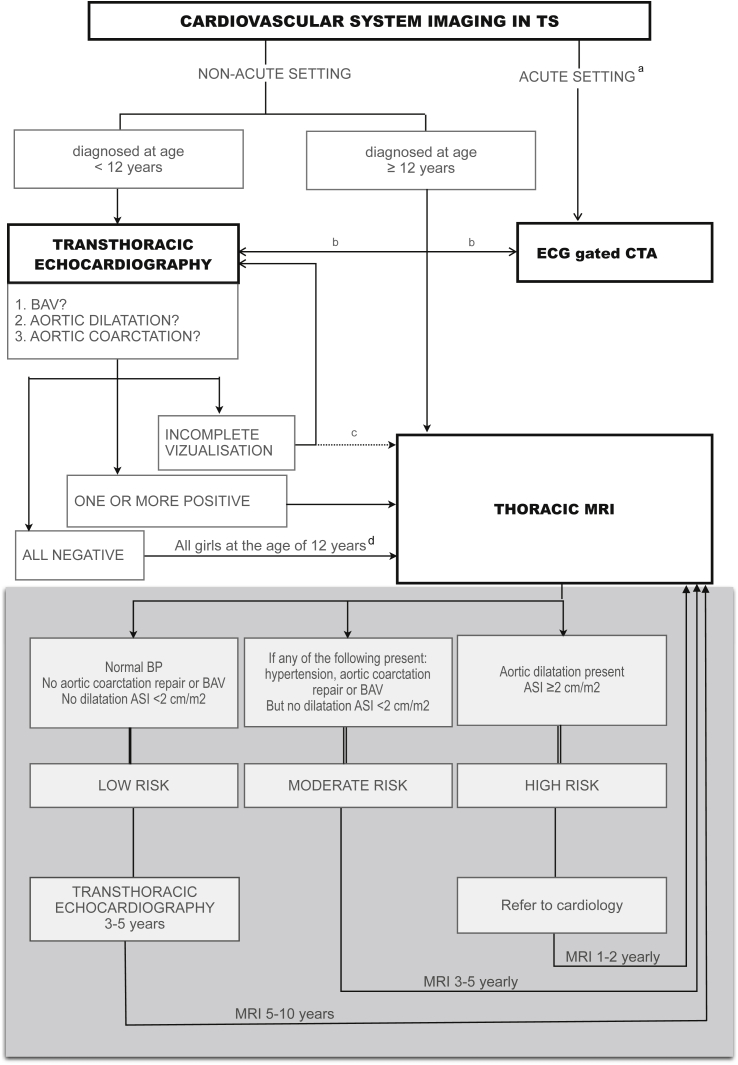
A summary of recommended cardiovascular system imaging in patients with TS. (The grey highlighted section is adapted with permission from Turtle et al.^8^) (a) When aortic dissection and/or coarctation is clinically suspected. (b) When MRI is not available or contraindicated. (c) When second transthoracic echocardiography is non-contributable. (d) When MRI can be without general sedation.

Transthoracic echocardiography is the imaging method of choice in the initial assessment of the cardiovascular system in the neonate, toddler, and young girl with TS. It provides an accurate and reliable non-irradiating assessment of the cardiovascular anatomy, including the root and the ascending part of the thoracic aorta and the entry of the pulmonary veins into the left atrium.^32^ However, in adolescents and adults, the shape of the thorax in TS can lead to suboptimal echocardiography images.^39^ In addition, transthoracic echocardiography is also limited in visualising the left upper pulmonary vein, and poor at visualisation of the thoracic aorta distal to the ascending portion.^12,63,64^ It has been generally accepted that a thorough characterisation of the cardiovascular anatomy is necessary at the time of TS diagnosis.^58^ Transthoracic echocardiography enables identification of the life-threatening cardiovascular disorders and their complications in the majority of children with TS. Although transthoracic echocardiography remains a standard follow-up investigation in many centres due to its availability and relatively low cost, in the case of incomplete or suboptimal visualisation of cardiovascular anatomy with echocardiography, or in the case of clinical indication, MRI should be performed.^22,39^ The importance of screening in younger age groups needs to be stressed because aortic dilatation can occur in girls as young as 5 years of age.^13,65,66^ At the age of 12 years, when most children would tolerate an MRI procedure without sedation, thoracic MRI should be performed.^67^ An imaging protocol has been previously proposed by Turtle et al.^8^ (Fig 7), which should be implemented at the transition to adulthood (around 16 years of age).

MRI provides a ionising radiation free and non-invasive assessment of cardiovascular anatomy with good visualisation of the heart chambers, myocardium, and the valves and a clear visualisation of the entire thoracic aorta, enabling recognition of the clinically and sonographically silent anomalies, such as a mild dilatation of the aorta or an elongation of the transverse aortic arch with kinking.^32,39^ As mentioned previously, MRI can supplement transthoracic echocardiography in cases of inadequate visualisation of the aortic valve leaflets and combination of both imaging methods yields diagnostic visualisation of the valve in almost all cases.^22^

In certain clinical conditions, there is a role for ECG-gated CT aortography in imaging of the acute aortic syndrome, and occasionally CT coronary angiography may be of use in the investigation of chest pain, particularly given the increased cardiovascular events in this population. ECG-gated CT angiography is the diagnostic method of choice in the setting of suspected aortic dissection in TS patients, clearly demonstrating the intimal flap, as well as the entry and re-entry sites and branch vessel involvement. This allows for prompt and accurate assessment on which to plan subsequent management strategies. Non-ECG-gated CT angiography can be used when ECG gating is not available, but it must be performed with the knowledge that small type A dissection flaps can be both under- and over-called due to movement artefact at the aortic root.^51^ In addition, the excellent spatial resolution and ease of multiplanar reformatting of CT angiography make it an excellent tool for assessing the anatomical structure of both the heart, pulmonary venous drainage, valve structure, and thoracic aorta, particularly where MRI is not available or contraindicated, although the associated radiation dose in a typically young population make this suboptimal for long-term follow-up.^68^

## Discussion

A review of the literature on TS has demonstrated that cardiovascular disorders are a major factor in the higher SMR of these patients compared to the general population. More troublesome is the early age at which TS patients can develop life-threatening cardiovascular complications. It is generally accepted that at the time of diagnosis of TS, echocardiography should be routinely performed as a part of a full cardiovascular evaluation. The review also demonstrates that thorough imaging of the aortic valve and the thoracic aorta are essential. Although, the specific timing of screening and follow-up are as yet undetermined, Fig 7 summarises our recommendations for a long-term cardiovascular imaging programme for patients with TS.

## Conclusion

Turner's syndrome is associated with high mortality from cardiovascular disorders. Awareness of the common findings in Turner's, in combination with a structured approach to imaging and follow-up, will provide maximum yield in terms of early identification and management of a wide range of potentially serious cardiovascular conditions.

## Acknowledgements

J.R. Weir-McCall is supported by the Wellcome Trust through the Scottish Translational Medicine and Therapeutics Initiative (Grant no. WT 085664) in the form of a Clinical Research Fellowship.

## Figures and Tables

**Table 1:** The imaging findings of the associated congenital anomalies and acquired diseases in Turner's syndrome.

System	Congenital anomaly or acquired disease	Frequency/risk	Occurs in combination with
Cardiovascular	Bicuspid aortic valve	30%^22^ (versus 1–2% in general population)^21,22^	Aortic coarctation, neck webbing^17^
	Aortic dilatation	32–42%^15,39,40^	BAV, aortic coarctation, hypertension or independently
	Aortic dissection	100-fold increased risk, 36 cases/100,000 patient years (versus 6/100,000 in general population)^9^	BAV, aortic coarctation, hypertension
	Aortic coarctation	10–12%^12,69^	BAV, neck webbing^17^
	Elongated transverse arch	49%^12^	
	Partial anomalous pulmonary venous return	13%^57^	
	Ischaemic heart disease	50%, appearing 6–13 years earlier than expected^20^	Arterial hypertension, hyperlipidaemia
Skeletal	Osteoporosis	10–50%^70–72^	Prolonged hypogonadism
	Bone fractures	5–45%^70,72^	Osteoporosis
	Cervical vertebral hypoplasia		
	Scoliosis	5–10%^73^	Growth hormone therapy
	Cubitus valgus	Up to 50%	
	Genu valgum	60%^74^	
	Short metacarpals and metatarsals		
	“Bayonet deformity” or Madelung's deformity		
Renal	Horseshoe kidney, duplex systems, and long posteriorly rotated kidneys	33–38%^75,76^	
Reproductive	Gonadal dysgenesis	90% require hormone-replacement therapy^4^	

**Table 2:** Summary of studies looking at MRI assessment of aortic dilatation and dissection in Turner's syndrome.

Study	No	Sequence	Measurements	Findings
Aortic dilatation				
Dawson-Falk 1992^77^	40	ECG-gated T1W “black blood” TSE sequence	Axial stack through aorta with diameter measured on slice with most dilated aorta	Aortic dilation in 12.5% (indexed diameter >95th CI based on CT values). 80% of these were only seen on MRI
Castro 2002^78^	15	ECG-gated T1W “black blood” TSE sequence	Axial stack through aorta with diameter measured 1 cm above aorta root	Ascending aortic dilation in 40% (indexed diameter >95th CI based on CT values); 26.7% had AD:DD >1.5
Ostberg 2004^39^	115	No details	Ascending and descending aorta at level of right pulmonary artery	Ascending aorta dilated in 33% using MRI criteria (AD:DD >1.5), but only 7% met both MRI and echocardiography criteria for dilatation. Dilated root associated with age and BAV
Chalard 2005^65^	21	ECG-gated T1W “black blood” TSE sequence	Two axial slices producing four measurements	19% (*n* = 5) had ascending aortic dilatation (not defined)
Ilyas 2006^79^	17	ECG-gated T1W “black blood” TSE sequence	Transverse plane (no further information provided)	Article focused on seven case series. *n* = 1 with aortic dilatation, which developed and progressed during imaging follow-up
Bondy 2006^80^	101	ECG-gated T1W “black blood” TSE sequence	Axial slice at level for ascending and descending aorta at level of RPA	Growth hormone has no effect on indexed aortic size
Matura 2007^40^	166	ECG-gated T1W “black blood” TSE sequence	Ascending and descending aorta at level of RPA	32% have ASI >2. 9.5% have AD diameter >mean +2 SD of control population, 32% have ASI >2, 45% have AD:DD >1.5
Lanzarini 2007^32^	59		Five levels within the thoracic aorta and one in the proximal abdominal aorta	Good correlation between echo and CMR in ascending aorta, however poorer correlation in rest of aorta.
Sachdev 2008^22^	15	ECG-gated T1W “black blood” TSE sequence	Four locations in ascending aorta (annulus, sinus, STJ, ascending aorta)	Aortic root dilated (indexed diameter >mean +2 SD) in 25% of patients with BAV compared with 5% of TAV
Cleeman 2010^81^	41	3D SSFP at diastole	Nine locations in thoracic aorta	No dilatation in mean aortic diameter, however aortic dilatation (indexed diameter >mean +2 SD of control group) was present in *n* = 5 in at least one of the nine measured locations. Using AD:DD, dilation was present in 28% of TS and 32% of controls.
Hjerrild 2010^41^^,^^a^	102	3D SSFP at diastole	Eight locations in thoracic aorta	23% had aortic dilation (indexed diameter >mean +2 SD) in at least one location, with dilation in ≥2 locations in 14%. In the latter group, 85% had BAV. Aortic diameter correlated with age, sex, BP, and presence of CoA and BAV.
Mortensen 2010^52^^,^^a^	99	3D SSFP at diastole	Eight locations in thoracic aorta	TS have 6.7X RR of ascending aortic dilation compared to the general population. Ascending aorta dilation associated with BAV and aortic coarctation and 45X monosomy.
Kim 2011^82^	51	MRA	Nine locations in thoracic aorta	Ascending aorta dilatation was common, with 30% with dilated aortic sinus (indexed diameter >mean +2 SD). 40.8% had AD:DD >1.5.
Mortensen 2011^24^^,^^a^	80	3D SSFP at diastole	Nine locations in thoracic aorta at baseline and 2 yrs follow-up	At a mean follow-up of 2.4 ± 0.4 yrs, increased dilatation was seen in the aortic sinus, sinotubular junction and mid-ascending aorta. Mean growth rate 0.1–0.4 mm/yr. BAV associated with more rapid growth rate than TAV (0.44 ± 0.57 versus 0.18 ± 0.61 mm/yr/m^2^)
Mortensen 2013^44^^,^^a^	102	3D SSFP at diastole	Eight locations in thoracic aorta at baseline, 2 yrs and 5 yrs	Significant growth seen in ascending but not descending aorta. Growth rates varied from 0.20 ± 0.34 to 0.38 ± 0.46 mm/yr for the three most proximal ascending aorta measurements. Age, CoA, BAV were associated with an accelerated growth while diastolic BP and hypertensive treatment were associated with slower growth
Aortic dissection				
Study	No	Age	Measurements	Findings
Carlson 2007^49^	2 (+85 literature review)	30.7 (4–64)	Echocardiography	15% had hypertension, 30% had congenital heart disease, 34% had both, 11% had no known risk factors. Assisted reproduction was present in 7/85, in this cohort there was an 86% mortality
Matura 2007^40^	3	49.3 (44–47)	MRI with black blood axial sequence at level of right pulmonary artery	All had ASI >2.5. Dissection occurred in 25% with AD >3.5cm, 33% of those with ASI >2.5, and only 3% with AD:DD >1.5. Annualised rate of 618/100,000 woman yrs
Carlson 2012^46^	20	31.5 (18–48)	Echocardiography in *n* = 15	Twenty dissections in 22 yrs. Type A dissection in 85%, type B in 15%. Mean ASI was 2.7 ± 0.6 cm/m^2^; 95% spontaneous dissections had a BAV, with 26% having an additional aortic pathology
Gravholt 2006^9^^,^^b^	18	35 (18–61)	Echocardiography in *n* = 10	Fourteen were 45X, 5 had BAV, and 5 had hypertension. Histology was available in 7 with 3 showing cystic medial necrosis. Estimated incidence of 36/100,000, compared with 6/100,000 in the general population (although the latter is not age matched)

a Same centre with overlapping populations.

b Included in Carlson literature review. Included separately as this is the only large-scale epidemiological review of dissection in TS.
